# Thoracic lymphatic anomalies in patients with univentricular hearts: correlation of morphologic findings in isotropic T2-weighted MRI with the outcome after fontan palliation

**DOI:** 10.3389/fcvm.2023.1145613

**Published:** 2023-05-09

**Authors:** Anja Hanser, Michael Hofbeck, Melanie Hofmeister, Petros Martirosian, Andreas Hornung, Michael Esser, Fritz Schick, Renate Kaulitz, Jörg Michel, Konstantin Nikolaou, Jürgen Schäfer, Christian Schlensak, Ludger Sieverding

**Affiliations:** ^1^ Department of Pediatric Cardiology, University Children's Hospital, University of Tübingen, Tübingen, Germany; ^2^ Section on Experimental Radiology, University Hospital of Tübingen, Tübingen, Germany; ^3^ Department of Diagnostic and Interventional Radiology, University Hospital of Tübingen, Tübingen, Germany; ^4^ Department of Cardiothoracic and Vascular Surgery, University Hospital of Tübingen, Tübingen, Germany

**Keywords:** TCPC, fontan circulation, thoracic and cervical lymphatic abnormalities, T2-weighted CMR, univentricular cardiac disease, failing fontan circulation

## Abstract

**Objectives:**

In this study we examined the correlation between the extent of thoracic lymphatic anomalies in patients after surgical palliation by total cavopulmonary connection (TCPC) and their outcome in terms of clinical and laboratory parameters.

**Materials and methods:**

We prospectively examined 33 patients after TCPC with an isotropic heavily T2-weighted MRI sequence on a 3.0 T scanner. Examinations were performed after a solid meal, slice thickness of 0.6 mm, TR of 2400 ms, TE of 692 ms, FoV of 460 mm, covering thoracic and abdominal regions. Findings of the lymphatic system were correlated with clinical and laboratory parameters obtained at the annual routine check-up.

**Results:**

Eight patients (group 1) showed type 4 lymphatic abnormalities. Twentyfive patients (group 2) presented less severe anomalies (type 1–3). In the treadmill CPET, group 2 reached step 7.0;6.0/8.0 vs. 6.0;3.5/6.8 in group 1 (*p* = 0.006*) and a distance of 775;638/854 m vs. 513;315/661 m (*p* = 0.006*). In the laboratory examinations, group 2 showed significantly lower levels of AST, ALT and stool calprotectin as compared to group 1. There were no significant differences in NT-pro-BNP, total protein, IgG, lymphocytes or platelets, but trends. A history of ascites showed 5/8 patients in group 1 vs. 4/25 patients in group 2 (*p* = 0.02*), PLE occurred in 4/8 patient in group 1 vs. 1/25 patients in group 2 (*p* = 0.008*).

**Conclusion:**

In the long-term follow-up after TCPC, patients with severe thoracic and cervical lymphatic abnormalities showed restrictions in exercise capacity, higher liver enzymes and an increased rate of symptoms of imminent Fontan-failure such as ascites and PLE.

## Key points

– This prospective study in 33 patients with univentricular hearts after total cavopulmonary connection (TCPC) shows that the severity of lymphatic abnormalities of the neck and thorax is correlated with clinical and laboratory parameters.

– Patients after TCPC with type 4 classification of lymphatic anomalies showed increased markers for failing Fontan including PLE, which is prognostically unfavourable.

## Introduction

Since the introduction of the Fontan operation in 1968 TCPC has become the treatment of choice for separation of pulmonary and systemic circulations in patients with functionally univentricular hearts (1–3). The worldwide number of patients increases annually and was estimated to be 50 000–70 000 in 2018 (3, 4). The 30-years survival after surgical Fontan completion is estimated > 80% (3–5). Since these patients develop late sequelae including hepatic fibrosis, protein-losing enteropathy, plastic bronchitis and ventricular dysfunction, morbidity and mortality remain high (6). The impairment of the lymphatic system, caused by increased venous pressure that is associated with alterations in starling forces, can lead to related complications. Understanding the physiology and pathophysiology of the lymphatic system and its treatment of complications are subject of current research, which Dori et al. in particular are driving forward with their work (7–10). They established a score for gradation of lymphatic abnormalities in the neck and thorax. Patients with severe thoracic lymphatic abnormalities were summarized under type 4 and had a significantly worse postoperative outcome after TCPC completion including prolonged duration of effusions, failure of Fontan circulation requiring Fontan takedown, need for transplant and higher 3-years mortality (11). Heavily T2-weighted magnetic resonance sequences have been introduced as a noninvasive technique to visualize the lymphatic system (10, 12, 13). In this study we investigated the incidence of anatomic variations of the thoracic lymphatic channels in patients after TCPC using a heavily T2-weighted 3D Fast Spin Echo (FSE) sequence and correlated the results with laboratory and clinical parameters (14). The aim of this study was to explore which symptoms are associated with type 4 lymphatic abnormalities.

### Materials and methods

This prospective study was approved by the local ethics committee with the project number (873/2017BO1). All subjects provided written informed consent. Written informed consent was obtained from the patients or their legal representatives.

### Study cohort

We performed a prospective study including thirty-three consecutive patients [Mdn (Q1;Q3) = 19.8 years (14.6;30.2)] patients with a history of TCPC [follow-up 14.3 years (9.7;24.9)] who underwent cardiac MRI between August 20, 2018, and July 5, 2021. Cardiac MRI every 5 years is part of our routine follow-up in patients with univentricular hearts following TCPC. All patients who qualified for MRI during the study period were offered MRL (magnetic resonance lymphangiography). Exclusion criteria were implanted pacemakers and absence of consent of the patient and/or parents. The results of the MRI examination were correlated with other data from the annual check-up such as medical history, physical examination, echocardiography, sonography of the abdomen and hemodynamic data derived by cardiac catherization. Exercise tests were performed on the treadmill according to the protocol of the German Society of Pediatric Cardiology (15). This treadmill protocol includes an increase in speed of 0.5 km/h and an increase in gradient of 3% to a maximum of 21% every 1.5 min. Each increase is termed step.

### MR-Examination

Subjects were examined on a 3.0 T MAGNETOM Prisma^Fit^ MRI scanner (Siemens Healthcare, Erlangen, Germany) in supine position. Our protocol included a routine cardiac MRI examination and in addition a coronal Half-Fourier Acquisition with Single Shot Turbo Spin Echo (HASTE) sequence with breath-holding. For MR lymphangiography a 3D FSE sequence was applied using respiratory navigator gating with following parameters: isotropic voxel size 1.2 × 1.2 × 1.2 mm^3^, interpolated to 0.6 × 0.6 × 0.6 mm^3^, 224 slices per slab, slab thickness 27 cm, repetition time 2,400 ms, long echo time 692 ms, field of view 460 × 460 mm, matrix size 384 × 384. Imaging of the neck, chest and abdomen was performed in all patients. All patients were asked to eat a high-fat breakfast and were offered 200 ml of cream (fat content 60 g) 3 h before the MRI-scan to stimulate the lymphatic flow and thereby to improve image quality (14). In order to reduce undesired signals from the stomach, the volunteers drank 200 ml of pineapple juice prior to the MR examination. Pineapple juice reduces undesired signals from stomach due to shortening of the T2 relaxation time (16).

### Image analysis

MR lymphangiograms were reconstructed using targeted maximum-intensity projection (MIP) with a slab thickness of 30 mm. The lymphangiograms were evaluated in accordance by one experienced radiologist (M.E. with 6 year of experience in MRI) and two experienced pediatric cardiologists (A.H.^1^, A.H.^2^ with 5 and 11 of years of experience in cardiac MRI), all with special expertise in cardiovascular MRI in CHD. For evaluation of the cervical and thoracic lymphatic channels the score according to Biko et al. was applied (11). The examiners were blinded for the clinical data of the patients, read all MRL studies together and classified the findings in consensus (14). Type 1 shows minimal supraclavicular increased signal intensity. Type 2 is characterized by increased signal intensity within bilateral supraclavicular region without extension into the mediastinum. Type 3 shows presumed lymphatic channels within the neck with extension of lymphatics within the mediastinum. Type 4 is characterized by increased abnormal signal intensity in the bilateral supraclavicular regions extending into the mediastinum and with interstitial pattern into the lungs (11).

### Statistical analysis

Statistical analysis was conducted using SPSS, version 28.0 (IBM Corp., Armonk, NY, USA). Distribution of demographic characteristics, cardiac MRI parameters, echocardiographic parameters and laboratory parameters were evaluated by two-sided *U*-test. Values of each variable were compared and expressed by median (Q1;Q3). A two-sided Chi-square test was used for comparison of clinical variables. A *p*-value of less than 0.05 indicated a statistically significant difference.

## Results

### Patients demographics, MRI scan and clinical history

**Figure 1:**
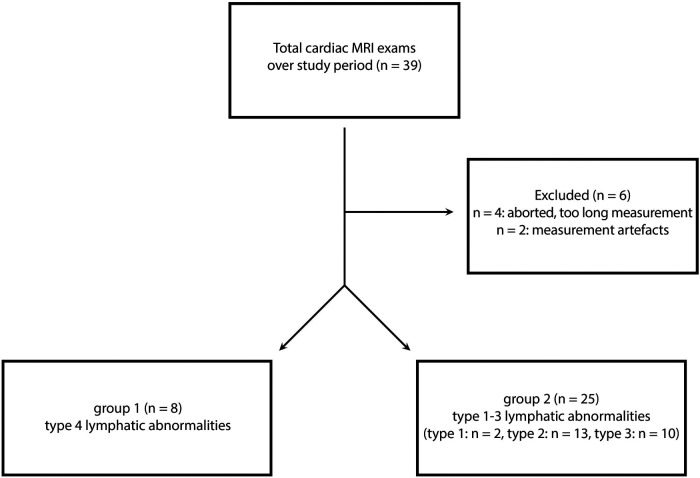
Flow diagram of study cohort.

**Figure 2:**
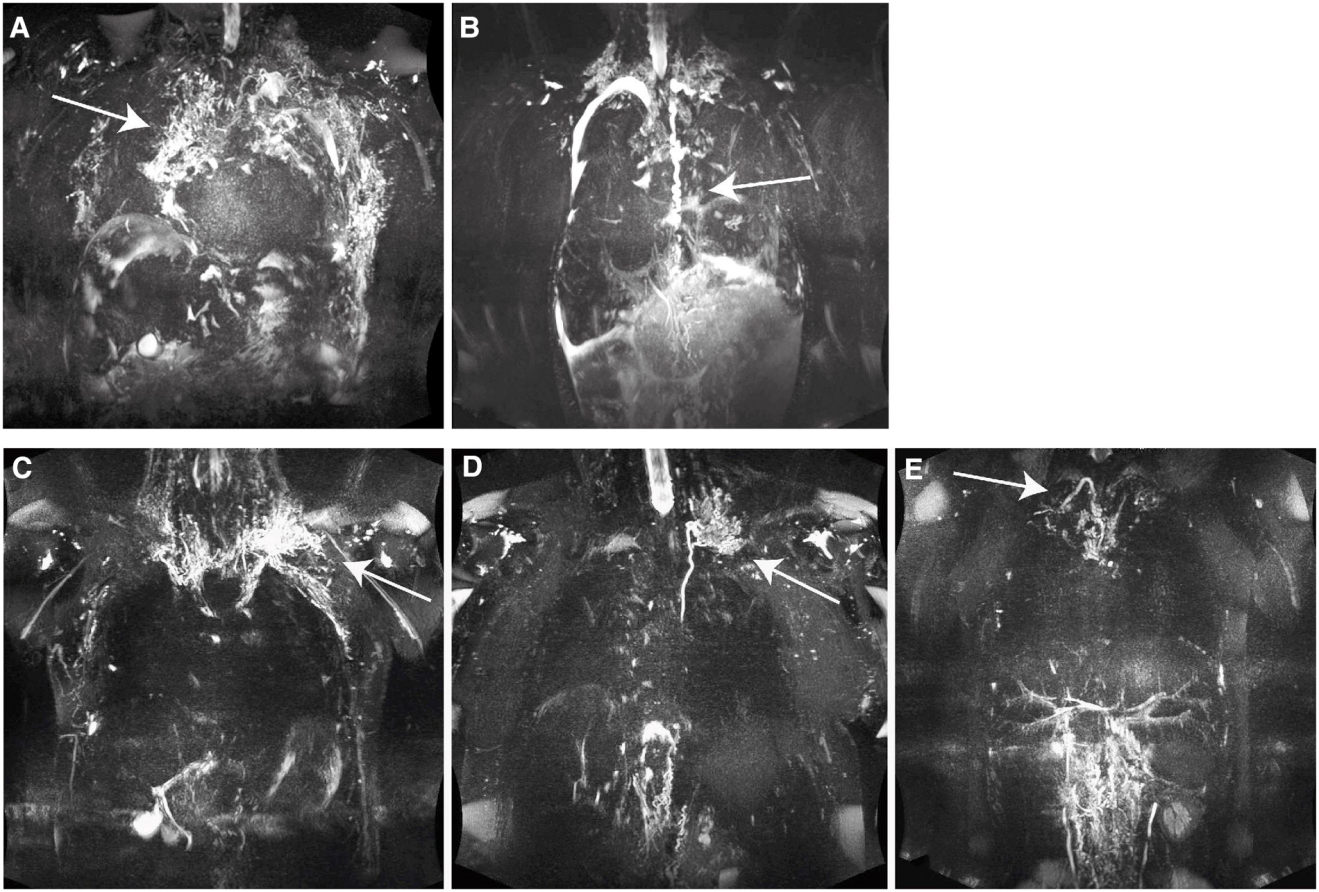
(**A**) classification type 4: patient 2, 19 years, male, hypoplastic left heart syndrome (HLHS), extracardiac conduit 20 mm, NYHA 3, PLE, pleural effusion, lymphatic abnormalities type 4 having abnormal supraclavicular lymphatics with extension both into the mediastinum and interstitial pattern into the lung parenchyma (arrow); MIP 30 mm. (**B**) Classification type 4: patient 31, 13 years, female, double inlet left ventricle (DILV), extracardiac conduit 18 mm, NYHA 4, PLE, pleural effusion, lymphatic abnormalities type 4 having abnormal supraclavicular lymphatics with extension both into the mediastinum and interstitial pattern into the lung parenchyma, tortuous duct (arrow); MIP 30 mm. (**C**) classification type 3: patient 17, 31 years male, pulmonary atresia with an intact ventricular septum, hypoplastic right heart, intraatrial conduit, NYHA 1, no PLE, lymphatic abnormalities type 3 having abnormal supraclavicular region with extension to the mediastinum (arrow); MIP 30 mm. (**D**) classification type 2: patient 28, 32 years, male, tricuspid atresia, hypoplastic right heart, intraatrial tunnel, NYHA 2, no PLE, lymphatic abnormalities type 2 having abnormal increased lymphatic channels within the supraclvicular region without extension to the mediastinum (arrow); MIP 30 mm. (**E**) classification type 1: patient 1, 20 years, male, atrioventricular septal defect, malposition of the great arteries, right isomerism, NYHA 1, no PLE, lymphatic abnormalities type 1 having little or no presumed lymphatic channels within the supraclavicular region and mediastinum (arrow); MIP 30 mm.

From August 2018 until July 2021, a total of 39 MRI investigations were performed in patients after TCPC at our tertiary referral center for children and adults with congenital heart disease (Figure 1). Six patients were excluded (4 patients aborted the MRI exam because of the duration of examination, 2 examinations were excluded because of excessive artefacts). A standard cardiac MRI protocol was performed followed by the heavily T2-weighted 3D FSE sequence. Thirty patients had a high-fat meal. Twenty-two of them followed up with an additional intake of 200 ml cream with 60 g fat content (3 of 8 patients (37.5%) in group 1 vs. 19 of 25 patients (76%) in group 2, Chi^2^(1) = 4.04, *p* = 0.082. The others refused the high-fat meal. Twenty-one patients tolerated it well, one patient reacted with vomiting. The FSE sequence was performed without interference in all patients. The acquisition time was 14:41 min (13:18;16:30). Eight patients (group 1) showed type 4 lymphatic abnormalities in the neck and thorax (Figures 2A, B). Twenty-five patients (group 2) showed lower grades abnormalities type 1-3 (Figures 2C–E) (11). Demographic data are shown in (Table 1). In group 1 (*n* = 8) six patients had an extracardiac conduit and two patients an intraatrial-lateral tunnel. In group 2 (*n* = 25) 12 patients had an extracardiac conduit, 11 patients an intraatrial-lateral tunnel and two patients an atriopulmonary anastomosis. The median diameter of the extracardiac conduits was 20.0 mm (18.0;21.0) in group 1 vs. 17 mm (16.0;19.5) in group 2, *p* = 0.075. The systemic ventricle was a left ventricle in 3 of 8 patients (37.5%) in group 1 whereas 18 of 25 patients (72%) had a left ventricle in group 2, *p* = 0.106.

**Table 1:** Demographic variables. SCPC = superior cavopulmonary connection, TCPC = total cavopulmonary connection.

	Group 1 (*n* = 8) median (Q1;Q3)	Group 2 (*n* = 25) median (Q1;Q3)	
Weight (kg)	**58.9** (30.5;64.1)	**61.0** (57.0;68.0)	*p* = 0.220
Size (m)	**1.63** (1.36;1.74)	**1.68** (1.61;1.78)	*p* = 0.138
Sex (f/m)	(**3**/**5)**	(**9**/**16)**	*p* = 0.940
Age MRI (y)	**16.8** (12.3;24.8)	**20.6** (16.3;31.1)	*p* = 0.074
Age Follow-up (y) (TCPC—MRI)	**10.4** (4.7;20.3)	**17.3** (12.3;25.5)	*p* = 0.044*
Age SCPC (m)	**11.3** (8.3;21.5) (mean ± SD: 14.5 ± 8.0)	**25.6** (9.8;63.3) (mean ± SD: 38.0 ± 31.6)	*p* = 0.081
Age TCPC (y) one stage Fontan	**3.1** (1.7;12.9) *n* = 1	**4.2** (3.2;6.2) *n* = 10	*p* = 0.220

Bold values are the median. *is statistically significant.

None of the patients required extracorporeal support postoperatively. None of the patients had additional cardiac operation during follow-up. The median number of cardiac catheter interventions such as stenting of the pulmonary arteries or occlusion of collaterals was 1.5 (0;3) in group 1 vs. 2.0 (0;1.5) in group 2, *p* = 0.254. The most recent cardiac catherization to MRI was within median 2.5 years (1.1;9.6), available in 28 patients, revealed a mean pulmonary artery pressures (PAPm) of 12.0 mmHg (11;17) in group 1 (*n* = 6) vs. 11.5 mmHg (8;13) in group 2 (*n* = 22), *p* = 0.214.

62.5% (5/8) of patients in group 1 received pulmonary vasodilative treatment with sildenafil as compared to 0% (0/25) of patients in group 2. There were no differences between the groups regarding the medication with acetylsalicylic acid, phenprocoumon, diuretics, ß-blockers, ACE inhibitors, amiodarone. Data regarding the history of diarrhea, ascites, PLE, plastic bronchitis and chylothorax are listed in Table 2.

**Table 2:** Frequency distribution of symptoms of PLE (protein-losing enteropathy), history of ascites, history of diarrhea, PB (plastic bronchitis) and chylothorax within the two groups.

	Group 1 *n* = 8	Group 2 *n* = 25	
PLE	4	1	Chi^2^ (1) = 9.98, *p* = 0.008*
Ascites (history of)	5	4	Chi^2^ (1) = 6.61, *p* = 0.020*
Diarrhoea (history of)	5	3	Chi^2^ (1) = 8.42, *p* = 0.010*
PB (history of)	2	1	Chi^2^ (1) = 3.23, *p* = 0.139
Chylothorax (history of)	4	5	Chi^2^ (1) = 2.75, *p* = 0.170

Bold values are the median. *is statistically significant.

Hepatomegaly was present in 37.5% (3/8) of patients in group 1 vs. in 8% (2/25) of patients in group 2, *p* = 0.078, Chi^2^(1) 4.10. The liver parenchyma showed no significant differences in parenchymal structure between both groups regarding homogeneity of parenchyma and surface nodularity. 50% (4/8) of patients in group 1 vs. 48% (12/25) of patients in group 2 showed inhomogeneous liver parenchyma, *p* = 1.0, Chi^2^(1) 0.1. Surface nodularity was present 50% (4/8) of patients in group 1 vs. 44% (11/25) of patients in group 2, *p* = 1.0, Chi^2^(1) 0.09.

There were no differences in echocardiographic parameters regarding AVVI, AI (Table 3). Cardiac MRI parameters including EF, normalized EDV also showed no differences (Table 3). Results for exercise capacity in treadmill CPET are shown in Table 3. Patients in group 2 achieved significantly longer distances and higher steps in these tests. However, there were no significant differences regarding the VO_2_max, O_2_ pulse and oxygen uptake efficiency slope (OUES).

**Table 3:** Echocardiographic, MRI and treadmill CPET variables.

	Group 1 (*n* = 8) median (Q1;Q3)	Group 2 (*n* = 25) median (Q1;Q3)	
**Echocardiographic parameters:**			
AVVI	**1** (0;1); *n* = 8	**1** (0;1); *n* = 25	*p* = 0.885
AI	**0** (0;0,8); *n* = 8	**0** (0;1); *n* = 25	*p* = 0.984
**MRI:**			
EF	**56** (44;67); *n* = 8	**47** (42;52); *n* = 25)	*p* = 0.150
EDVnorm (ml/m^2^)	**106** (85;113); *n* = 8	**94** (79.5;105.5); *n* = 8	*p* = 0.330
**Treadmill CPET:**			
Step	**6.0** (3.5;6.8); *n* = 8	**7** (6:8); *n* = 25	*p* = 0.006*
Distance (m)	**513** (315;661); *n* = 8	**775** (638;854); *n* = 25	*p* = 0.005*
VO_2_max (ml/kg/min)	**24.0** (22.5;25.8); *n* = 6	**24.7** (20.5;29.6); *n* = 25	*p* = 0.942
O_2_ pulse (ml)	**8.7** (5.8;11.1); *n* = 6	**9.4** (7.8;14.0); *n* = 25	*p* = 0.419
OUES [(L/min)/log10]	**1.76** (0.90;2.05); *n* = 6	**1.79** (1.43;2.54); *n* = 6	*p* = 0.314

EF ejection fraction; AVVI, atrioventricular valve insufficiency (0: none, 1: mild, 2: moderate, 3: severe); AI, aortic valve insufficiency (0:none, 1:mild, 2:moderate, 3: severe); EDVnorm, end-diastolic volume normalized; step according to the protocol of the German Society of Pediatric Cardiology (DGPK) (15); VO*_2_*max, peak oxygen consumption; OUES, oxygen uptake efficiency slope.

Bold values are the median. *is statistically significant.

**Figure 3:**
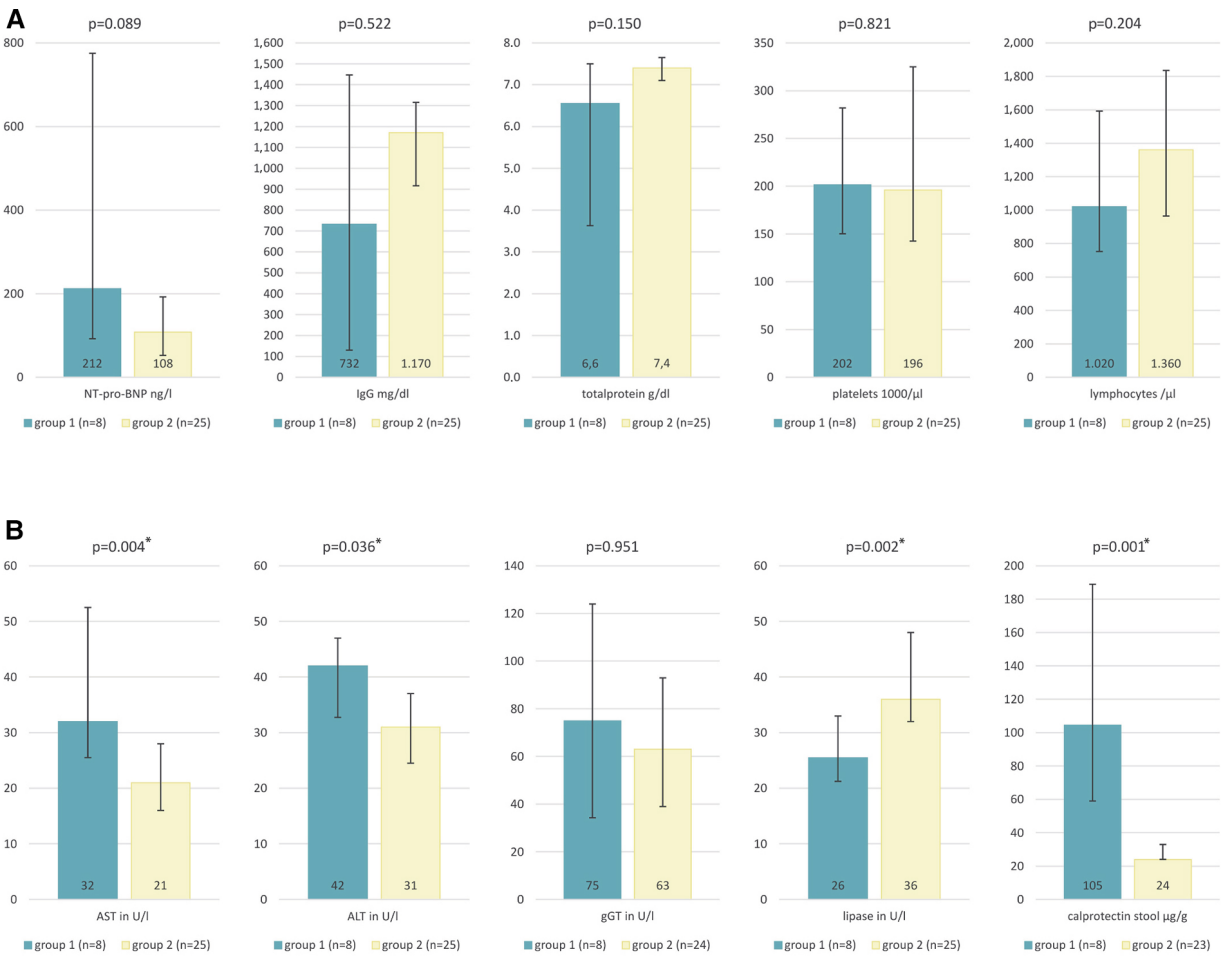
Laboratory parameters. (**A**) NT-pro-BNP group 1 212(92;775) ng/L vs. group 2 108(53;193) ng/L, IgG in group 1 732(130;1448) mg/dl vs. group 2 1170(917;1315) mg/dl, total protein in group 6.6(3.6;7.5) vs. group 2 7.4(7.1;7.7) g/dl, platelets in group 1 202(150;325) vs. group 2 196(143;282) 1000/µl, lymphocytes in group 1 1020(753;1593) vs. group 2 1360(965;1835) 1000/µl. (**B**) AST in group 1 32(26;53) vs. group 2 21(16;28) U/L, ALT in group 1 42(33;47) vs. group 2 31(25;37) U/L, *γ*GT in group 1 75(34;124) vs. group 2 63(39;93) U/L, lipase in group 1 26(21;33) vs. group 2 36(32;48) U/L, calprotectin in stool in group 1 105(59;189) vs. group 2 24(24;33)µg/g expressed as median (Q1;Q3).

Although not statistically significant, laboratory parameters for NT-pro-BNP tended to be higher in group 1, values for IgG, total protein, albumin and lymphocytes tended to be lower in group 1. Platelets showed similar values in both groups. (Figure 3A). Group 1 showed significantly higher levels for ALT and AST. Although not statistically significant, values for *γ*GT tended to be higher in group 1. Lipase and pancreatic amylase were measured significantly lower in group 1 vs. group 2. Values for calprotectin in stool were significantly higher in group 1 (Figure 3B). There were no differences in values for calprotectin in blood. The median of a1-antitrypsin in stool showed no differences, but in group 1 Q3 showed a higher range [group 1 0.1 mg/g (0.06;1.84) vs. group 2 0.12 mg/g (0.06;0.16)]. The spot urine samples showed no differences between the groups (Supplementary Material Table S1).

## Discussion

Functionally univentricular hearts encompass a variety of different complex congenital malformations. Due to the absence of a subpulmonary pumping chamber perfusion of the lungs is achieved by anastomoses of the superior and inferior vena cava with the pulmonary arteries (1–3). The driving force of the pulmonary perfusion is based to a large extent on the central venous pressure (CVP) which is always elevated in these patients as well as the diastolic function of the systemic ventricle (3, 17). While the Fontan circulation is functioning well in the majority of patients during the first decade of life, morbidity increases in the long-term follow-up (5, 18). Meanwhile there is evidence that elevated CVP in patients with Fontan circulation has major impact on the lymphatic system, resulting in impairment of the lymphatic system both in the thorax and in the abdomen (8, 12). Since these alterations have significant impact on quality of life and life expectancy, the lymphatic system has come into the focus of increasing diagnostic efforts during the recent years (7, 9, 12, 19). Heavily T2-weighted 3D FSE sequences have recently been introduced as a noninvasive technique for visualization of the lymphatic system (10, 13, 14). According to the data of our study this technique can be successfully employed for noninvasive imaging of the thoracic lymphatic system in the long-term follow-up of patients with functionally univentricular hearts. In a previous study we already demonstrated a high interobserver correlation in the assessment of the thoracic lymphatic system with this noninvasive method (intraclass correlation coefficient: pre 0.864 and 0.900; > 0.81) (14).

Little is known so far about the clinical relevance of morphologic changes in the lymphatic system and their correlation with clinical symptoms in patients following palliation by TCPC. Biko et al. examined patients following SPCP as the ideally second step of univentricular palliation (11). Patients with severe alterations of the lymphatic system (characterized by increased abnormal signal intensity in the supraclavicular regions extending into the mediastinum and with interstitial pattern into the lungs) showed significantly worse clinical outcomes following TCPC (11). To the best of our knowledge our study is the first one correlating findings of noninvasive imaging of lymphatic abnormalities in the neck and thorax with clinical findings in the long-term follow-up after TCPC (20). In accordance with Biko and Dori et al. we compared patients with pronounced alterations of the lymphatic system (group 1) with the patients with lower grades of lymphatic anomalies (group 2).

In contradiction to the assumption that the Fontan circulation gradually deteriorates over time patients with more pronounced type 4 lymphatic abnormalities (group 1) had a significantly shorter median interval (10.4 years vs. 17.3 years) between completion of the TCPC and the MRI-study. This cannot be explained by a selection bias in favour of clinically symptomatic patients, since MRI was offered to all Fontan patients during this time period irrespective of clinical symptoms. Interestingly age at the time of SCPC and age at the time of TCPC were lower among patients with more pronounced lymphatic abnormalities however without reaching statistical significance. Ten patients in group 2 underwent the TCPC without preceding SCPC. The shorter follow-up period in patients with more significant lymphatic changes could be due to the fact that there was a tendency in the last two decades to perform the SCPC at a younger age. Several studies revealed that pulmonary artery growth decreases significantly following establishment of a non-pulsatile pulmonary circulation by creation of a cavopulmonary connection (17, 21–23). Impaired pulmonary artery growth due to non-pulsatile blood flow at an earlier age might result in increased pulmonary vascular resistance and CVP with subsequent backlog to the systemic venous and lymphatic system (23, 24). Further studies on larger numbers of patients will be required to clarify the fact, whether early establishment of cavopulmonary connections might be detrimental in the long-term outcome of these patients. Furthermore, it is not clear yet if specific congenital anatomical variants of the lymphatic system, especially in patients with univentricular hearts, might predispose patients to more severe alterations in the long-term follow-up (25, 26). Comparison of our groups regarding clinical symptoms revealed that 50% of patients in group 1 showed symptoms of PLE, as compared to 4% in group 2. This finding suggests that lymphatic abnormalities in these patients may not be restricted to the thorax but also include the abdomen. A history of ascites and diarrhea representing possible signs of impaired abdominal lymphatic circulation were observed more frequently in group 1. The incidence of plastic bronchitis in our patient group was too low to allow statistical comparison of both subgroups.

Regarding laboratory parameters there were no significant differences in NT-pro-BNP, total protein, IgG, lymphocytes or platelets. While these differences did not reach statistical significance the trends in our cohort are directed towards the changes seen in patients with Failing Fontan circulation and PLE (3). Group 1 patients showed significantly higher values for calprotectin in the stool with 105 µg/g vs. 24 µg/g. Calprotectin as a marker for inflammation gives a valid estimate of intestinal inflammation, comparable with inflammatory bowel disease (27, 28). Miranda et al. found significantly higher values of fecal calprotectin in Fontan patients with PLE (*n* = 5) than in Fontan patients without PLE (*n* = 18) (27). The elevated values for calprotectin in stool in group 1 of our cohort are in accordance with the increased incidence of PLE among these patients. The elevated values support the hypothesis that type 4 lymphatic abnormalities in the neck and thorax represent severe affection of the lymphatic system and represent markers for future problems of imminent Failing Fontan circulation. Results in echocardiographic and cardiac MRI parameters in our cohort revealed no differences between both groups. This finding is consistent with previous studies which were unable to detect early functional parameters defining patients with predisposition to a Failing Fontan circulation (24, 29). In our cohort patients of group 1 showed some significant differences on treadmill CPET as compared to patients of group 2. Patients with less pronounced lymphatic abnormalities were able to achieve significantly longer distances and higher steps in these tests. However, there were no significant differences regarding the VO_2_max, O_2_ pulse and oxygen uptake efficiency slope (OUES), but trends. While the former result would suggest a lower exercise tolerance in patients with more pronounced lymphatic abnormalities, we are unable to explain, the absence of a significant difference in VO_2_max, which is considered as an important parameter reflecting exercise tolerance. Possibly the relatively small number of patients in group 1 may have contributed to the absence of significant difference in this parameter. Two patients in group 1 with poor exercise tolerance, who reached only step 3 (distance 226 m and 290 m) of the treadmill protocol, did not tolerate a mask. Therefore no spirometric data were available in these patients. According to data from Gewillig et al. that patients with poorer Fontan circulation are characterized by a significantly decreased exercise capability (18). It is not possible to achieve an increase in cardiac output in the Fontan circulation under exercise through an increase in heart rate alone because of the absence of a subpulmonary ventricle. The cardiac output is determined to a large extent by the pulmonary vascular resistance (PVR) (18). Increasing PVR is frequently observed in the long-term follow-up after TCPC limiting the exercise capability of these patients. In our cohort we were unable to correlate the lymphangiographic and clinical findings with data from cardiac catherization since not all patients underwent invasive testing within 12 months of the cardiac MRI. However, the inferior clinical situation in patients of group 1 is reflected by the fact that 5/8 patients in this group received pulmonary vasodilative therapy contrary to none of the 25 patients in group 2 (17, 18, 30).

In summary patients with pronounced abnormalities of the thoracic and cervical lymphatic systems revealed more symptoms of an imminent deterioration of the Fontan circulation. According to these findings patients with TCPC and type 4 anomalies of the cervical and thoracic lymphatic system according to Biko et al. should be considered as high-risk patients for the development of a failing Fontan circulation requiring careful follow-up in specialized centers. Since there are no data yet on the timing of development of these changes of the lymphatic system, serial examinations will be required to describe the onset and the progress of these anomalies. Due to its noninvasive nature isotropic highly resolved 3D T2-weighted MRI appears to be a valuable tool to improve the understanding of these anomalies.

**Limitations** of the study are due to the relatively small number of patients. Examinations were performed at a single point in time and are unsuitable to describe the timing of development and progression of lymphatic abnormalities. In the study period all patients who had routine cardiac MRI for evaluation of TCPC were offered MRL making a selection bias unlikely. We are unable however to exclude some bias which might originate from a rather long follow-up period. While the results of this study do not imply a causal relationship between type 4 lymphatic abnormalities and restrictions in exercise capacity, these abnormalities seem to represent another marker indicating unfavourable Fontan hemodynamics.

## Conclusion

The heavily T2-weighted 3D FSE sequence is a noninvasive MRI technique which can be performed without contrast agent under free-breathing condition to visualize the thoracic and cervical lymphatic system. In the long-term follow-up after TCPC patients with severe thoracic and cervical lymphatic abnormalities showed restrictions in exercise tolerance and increased symptoms of compromised lymphatic circulation including PLE and ascites. Further studies are required to clarify the causality, onset and progress of these changes.

## Data Availability

The raw data supporting the conclusions of this article will be made available by the authors, without undue reservation.

## Supplementary material

Movie 1 patient 2, 19 years, male, hypoplastic left heart syndrome (HLHS), extracardiac conduit 20 mm, NYHA 3, PLE, pleural effusion, lymphatic abnormalities type 4 having abnormal supraclavicular lymphatics with extension both into the mediastinum and interstitial pattern into the lung parenchyma; MIP 30 mm.

Movie 2 patient 31, 13 years, female, double inlet left ventricle (DILV), extracardiac conduit 18 mm, NYHA 4, PLE, pleural effusion, lymphatic abnormalities type 4 having abnormal supraclavicular lymphatics with extension both into the mediastinum and interstitial pattern into the lung parenchyma, tortuous duct; MIP 30 mm.

Movie 3 patient 17, 31 years male, pulmonary atresia with an intact ventricular septum, hypoplastic right heart, intraatrial conduit, NYHA 1, no PLE, lymphatic abnormalities type 3 having abnormal supraclavicular region with extension to the mediastinum; MIP 30 mm.

Movie 4 patient 28, 32 years, male, tricuspid atresia, hypoplastic right heart, intraatrial tunnel, NYHA 2, no PLE, lymphatic abnormalities type 2 having abnormal increased lymphatic channels within the supraclavicular region without extension to the mediastinum; MIP 30 mm.

Movie 5 patient 1, 20 years, male, atrioventricular septal defect, malposition of the great arteries, right isomerism, NYHA 1, no PLE, lymphatic abnormalities type 1 having little or no presumed lymphatic channels within the supraclavicular region and mediastinum; MIP 30 mm.
